# Lamotrigine serum concentrations as longitudinal biomarkers for seizure risk prediction in pregnant women with epilepsy: a secondary analysis of the EMPiRE study

**DOI:** 10.1136/bmjno-2026-001547

**Published:** 2026-06-22

**Authors:** John Allotey, Fatima Junaid, Teresa Peréz, Valeria Rolle, María del Carmen Pardo, Javier Zamora, Shakila Thangaratinam

**Affiliations:** 1 Institute of Life Course and Medical Sciences, University of Liverpool, Liverpool, England, UK; 2 Department of Metabolism and Systems Science, University of Birmingham, Birmingham, England, UK; 3 Department of Obstetrics and Gynaecology, Birmingham Women’s Hospital, Birmingham, England, UK; 4 Department of Statistics and Data Science, Complutense University of Madrid, Madrid, Spain; 5 Instituto de Estadística y Ciencia de los Datos, Madrid, Spain; 6 Plataforma de Bioestadística y Epidemiología, Instituto de Investigación Sanitaria del Principado de Asturias, Avenida Hospital Universitario, Oviedo, Spain; 7 Department of Statistics and Operational Research, Complutense University of Madrid, Madrid, Spain; 8 Hospital Universitario Ramón y Cajal, Madrid, Community of Madrid, Spain; 9 Liverpool Women’s NHS Foundation Trust, Liverpool, England, UK

**Keywords:** OBSTETRICS, EPILEPSY

## Abstract

**Background:**

Serum lamotrigine concentrations fall during pregnancy, and therapeutic drug monitoring is often used to guide dose adjustment. We do not know whether serial antenatal measurements improve prediction of seizure risk in pregnant women with epilepsy.

**Objective:**

To evaluate whether changes in maternal serum lamotrigine concentration levels are associated with seizure occurrence in pregnancy and whether serial measurements improve prediction of seizure risk beyond baseline clinical factors.

**Design:**

Secondary analysis of data from the AntiEpileptic drug Monitoring in PREgnancy (EMPiRE) study, a multicentre, double-blind randomised trial nested within a prospective cohort, using joint longitudinal-survival modelling.

**Setting:**

Fifty maternity units in the UK between November 2011 and May 2015.

**Methods:**

We included pregnant women with epilepsy taking lamotrigine (monotherapy or polytherapy) at baseline and who had a baseline and at least one follow-up serum lamotrigine concentration measured before a seizure or up to 6 weeks postpartum. The primary outcome was time to first seizure during pregnancy or within 6 weeks postpartum. We used joint longitudinal-survival models to assess the association between serial lamotrigine concentrations and seizure occurrence, adjusting for baseline clinical predictors from the validated EMPiRE seizure risk model. Associations were expressed as hazard ratios (HRs) with 95% confidence intervals (CIs).

**Results:**

Of 560 women recruited to the EMPiRE trial, 183 women contributed data; 46 women had seizures in pregnancy. Longitudinal serum lamotrigine concentrations were not associated with seizure occurrence (HR 0.421, 95% CI 0.157 to 1.133, p=0.087). Baseline lamotrigine daily dose was the only independent predictor of seizure (HR 1.005, 95% CI 1.003 to 1.007, p<0.001). The model based on serum lamotrigine levels had poor discrimination with an AUC ranging from 0.46 to 0.55 throughout pregnancy.

**Conclusion:**

Longitudinal serum lamotrigine concentrations do not appear to predict seizure occurrence in pregnancy. Seizure risk was better explained by baseline lamotrigine dose.

**WHAT IS ALREADY KNOWN ON THIS TOPIC**

- Serum lamotrigine concentrations fall during pregnancy in women with epilepsy because drug clearance increases.
- Some small studies, with variable design and moderate to high risk of bias, have suggested that falling lamotrigine levels may increase seizure risk during pregnancy.
- Guidance on routine monitoring of lamotrigine levels in pregnancy is inconsistent, and evidence for benefit is limited.
- No previous study has evaluated whether serial serum lamotrigine measurements improve prediction of seizure risk during pregnancy.

**WHAT THIS STUDY ADDS**

- In this secondary analysis of the AntiEpileptic drug Monitoring in PREgnancy (EMPiRE) study, serial serum lamotrigine measurements during pregnancy were not associated with seizures and did not improve prediction of seizure risk in pregnant women with epilepsy.
- Baseline lamotrigine dose was the only independent predictor of seizure risk.

**HOW THIS STUDY MIGHT AFFECT RESEARCH, PRACTICE OR POLICY**

- These findings provide limited support for routine monitoring of lamotrigine levels during pregnancy to predict seizures.
- Clinical management should continue to prioritise seizure history, adherence and clinical assessment.
- Future research should identify whether some groups of pregnant women with epilepsy may still benefit from therapeutic drug monitoring.

## Introduction

 Seizures are the second-leading indirect cause of maternal deaths.^1^ One-third of pregnant women with epilepsy experience changes in seizure frequency or experience seizures after having been seizure-free.^2^,^4^ Seizures in pregnancy increase the risk of maternal complications, including injury and sudden unexpected death, as well as adverse perinatal outcomes such as preterm birth and fetal growth restriction, and can also affect daily social life.^5 6^ Effective seizure control in pregnancy is therefore critical for maternal and fetal safety. This is generally achieved through antiseizure medications alongside strategies to minimise seizure triggers and other risks.^7^

Among antiseizure medications, lamotrigine is one of the most commonly prescribed drugs for women of childbearing age.^8^ Although reference ranges for therapeutic levels of lamotrigine exist, the optimal level needed for seizure control varies between individuals.^9 10^ Pregnancy alters the pharmacokinetics of many antiseizure medications, resulting in increased drug clearance and lower serum concentrations.^11^,^13^ There are concerns that decreases in serum lamotrigine levels during pregnancy may increase the risk of seizures.^14 15^ The American Academy of Neurology recommends routine therapeutic drug monitoring, where antiseizure medication doses are adjusted to maintain pre-pregnancy plasma levels throughout pregnancy.^16 17^ In the UK, guidelines from the National Institute for Health and Care Excellence recommend therapeutic drug monitoring for women considered at high risk of seizure deterioration but acknowledge that the evidence base is limited and mainly observational.^18^ Both the Royal College of Obstetricians and Gynaecologists and the Scottish Intercollegiate Guideline Network do not advocate routine therapeutic drug monitoring in pregnant women with epilepsy due to a lack of evidence regarding clinical utility, and instead advise monitoring in individual cases, for example, to assess adherence or toxicity.^7 19^

Evidence on the clinical utility of therapeutic drug monitoring in pregnancy is limited.^13 14 20^ The AntiEpileptic drug Monitoring in PREgnancy (EMPiRE) study found routine monitoring of antiseizure medications to be associated with increased drug exposure in newborns.^20^ Whether changes in serum antiseizure medication levels predict seizure risk in pregnancy or provide a basis for treatment adjustment remains unclear. We therefore analysed data from women on lamotrigine monotherapy or polytherapy enrolled in the EMPiRE study to evaluate the association between changes in maternal serum lamotrigine levels and seizures, and to assess whether serial measurements added prognostic value beyond established baseline clinical predictors. This is a biomarker evaluation study rather than development of a prediction model.

## Methods

### Study design and population

We undertook secondary analysis of data from the EMPiRE study, a prospective, multicentre, double-blind randomised trial nested within a cohort study conducted across 50 UK maternity units between November 2011 and May 2015.^20^ Women with a confirmed diagnosis of epilepsy, less than 24 weeks’ gestation, and receiving one of four antiseizure medications (lamotrigine, carbamazepine, levetiracetam or phenytoin) were eligible for monthly serum drug measurements during pregnancy and up to 6 weeks postpartum. Baseline serum concentration was the reference level used to assess changes during pregnancy. As pre-pregnancy levels were rarely available, the first level obtained after enrolment was used. Women whose serum drug level fell by more than 25% from baseline were randomised to therapeutic drug monitoring, where clinicians received serum levels and could adjust drug dose accordingly, or to clinical monitoring, where dose adjustments were based on clinical assessment alone. Women with stable serum drug levels formed the non-randomised cohort. For this analysis, we included all women in the EMPiRE study who were receiving lamotrigine, either as monotherapy or polytherapy at baseline, and who had baseline and at least one follow-up serum drug measurement before a seizure or before 6 weeks postpartum. The aim was to assess whether longitudinal lamotrigine concentrations were associated with seizure occurrence, and whether they added prognostic value beyond baseline factors included in the EMPiRE prediction model.

### Variables and outcomes

We used the same baseline predictors (co-variates) as the validated EMPiRE prediction model for seizure occurrence in pregnant women with epilepsy.^21^ The model was developed using baseline maternal and pregnancy characteristics to estimate individual seizure risk and included age at first seizure (years), history of learning difficulty or mental health disorder (yes/no), baseline seizure classification (tonic-clonic, non-tonic-clonic and unspecified), number of seizures in the 3 months before pregnancy (tonic-clonic and non-tonic-clonic), gestational age at baseline (weeks), history of hospital admission for seizures in a previous pregnancy (yes/no) and baseline daily dose of lamotrigine (mg/day). In addition, we included serum lamotrigine concentrations (mg/l), which were measured monthly during pregnancy and up to 8 weeks postnatal. The primary outcome was time to first seizure within pregnancy or up to 6 weeks postpartum.

### Statistical analysis

We compared baseline characteristics between women who experienced a seizure during pregnancy or within 6 weeks postpartum and those who did not using Student’s t-test for continuous variables and a χ2 test or Fisher’s exact test for categorical variables. The normality was assessed visually using Q–Q plots. We assumed missing data were missing at random (MAR) and imputed values using multiple imputation by chained equations. We used predictive mean matching for continuous variables and logistic regression for binary variables. We generated five imputed datasets incorporating both baseline and outcome variables and selected the dataset with the most similar density function to the observed data.

To evaluate the association between longitudinal serum lamotrigine concentrations and time to first seizure, we used joint modelling with a restricted maximum likelihood (REML) estimation. Joint modelling combines repeated biomarker measurements with time-to-event data, allowing us to estimate the dynamic effect of lamotrigine concentrations on seizure risk while adjusting for baseline covariates.^22 23^ We followed a three-step modelling framework. First, we fitted a linear mixed-effects model to describe how lamotrigine concentrations changed over time, adjusting for baseline covariates. Second, we developed a Cox proportional hazards model for time to first seizure, including baseline covariates with p<0·15 in univariable screening, and finally, we combined the longitudinal and survival submodels into a joint model, using splines to approximate the baseline hazard function. We also fitted additional models using relative changes from baseline, expressed as both absolute differences and concentration ratios. We checked model assumptions using residual plots and assessed multicollinearity among predictors. The proportional hazards assumption was assessed using Schoenfeld residuals, both graphically and through formal testing.^24^

Candidate variables for inclusion in the multivariable model were initially identified through univariable analyses, with variables showing an association with the outcome at a threshold of p<0.20 considered for inclusion. These variables were then entered into the multivariable model. Model refinement was subsequently performed using a combination of the Akaike Information Criterion (AIC) and the likelihood ratio test.

We performed internal validation using fivefold cross-validation. We randomly split the dataset into five disjoint sub-samples, and in an iterative process, each sub-sample served once as the validation cohort while the remaining four sub-samples were used to fit the joint model. We calculated the time-dependent area under the receiver operating characteristic curve (AUC) to evaluate model discrimination within the joint longitudinal survival modelling framework, defined as the ability to distinguish women who would experience a seizure within 1 week from those who would not, using all available data up to that time. Details of model development and assumptions are provided in online supplemental appendix A. All analyses were performed using R software, with the packages mice for imputation, jm and survival for the statistical modelling and dplyr for data management.^25^,^28^

### Patient and public involvement

Patients and the public were involved in the planning, conduct and reporting of the original EMPiRE study. There was no new involvement in this secondary analysis.

### Ethics

The West Midlands UK National Research Ethics Committee approved the EMPiRE study (11/WM/0164), written consent was obtained from participants, and the study details can be accessed at https://www.journalslibrary.nihr.ac.uk/hta/HTA22230.^20^ Use of anonymised data for the secondary analysis did not require further review by an ethics committee. We reported our study in line with TRIPOD (Transparent Reporting of a Multivariable Prediction Model for Individual Prognosis or Diagnosis) recommendations.

## Results

The EMPiRE study recruited 560 pregnant women with epilepsy. Of the 306 who were taking lamotrigine as monotherapy or polytherapy with carbamazepine, levetiracetam or phenytoin at baseline, 282 had both baseline and follow-up serum data; 183 women had at least one serum lamotrigine measurement before a seizure or within 6 weeks postpartum and were included in this analysis (online supplemental appendix B).

### Characteristics of the included women

The mean maternal age was 29.9 years (SD 4.3) in women without seizures and 29.5 years (SD 5.4) in those with seizures. Body mass index, gestational age at baseline, seizure type and proportion of women with a history of mental illness were similar in both groups. About half of the women were nulliparous, with no difference in parity between the groups. Women who had seizures were more likely to have experienced any type of seizure in the 3 months before pregnancy. The mean age at first seizure was slightly younger in women with seizures (18.5 years, SD 6.1) than in those without (20.3 years, SD 7.8), although this difference was not statistically significant. Daily dose of lamotrigine at baseline was higher in women with seizures (329 mg, SD 182) compared with those without seizures (219 mg, SD 121) (table 1).

**Table 1:** Baseline characteristics of women taking lamotrigine with and without seizures and the proportion with missing data.

Characteristics	No seizure	Seizure	p-value	Missing
	(n=137)	(n=46)		
Maternal age (years)	29.9 (4.3)	29.5 (5.4)	0.663	0 (0%)
Body mass index (kg/m^2^)	26.3 (5.5)	26.1 (6.8)	0.848	3 (1.6%)
Parity – n (%)			0.391	0 (0%)
Multiparous	57 (41.6%)	23 (50.0%)		
Nulliparous	80 (58.4%)	23 (50.0%)		
Gestational age at baseline (days)	114 (31.5)	116 (24.1)	0.684	0 (0%)
Seizure type			0.178	0 (0%)
Non-tonic-clonic	63 (46.0%)	28 (60.9%)		
Tonic-clonic	69 (50.4%)	16 (34.8%)		
Unspecified	5 (3.6%)	2 (4.3%)		
Seizures within 3 months pre-pregnancy				
Tonic-clonic			0.010	40 (21.9%)
0	106 (96.4%)	27 (81.8%)		
1	3 (2.7%)	5 (15.2%)		
2 or more	1 (0.9%)	1 (3.0%)		
Non-tonic-clinic			0.008	0 (0%)
0	130 (94.9%)	37 (80.4%)		
1	2 (1.5%)	4 (8.7%)		
2 or more	5 (3.6%)	5 (10.9%)		
Age at first seizure (years)	20.3 (7.8)	18.5 (6.1)	0.131	13 (7.1%)
History of mental illness (yes)	9 (6.8%)	3 (6.7%)	0.999	5 (2.7%)
Daily dose of lamotrigine at baseline (mg)	219 (121)	329 (182)	<0.001	0 (0%)

P-values come from Students’ t-test for continuous variables and χ2 or Fisher test for categorical variables. Numbers are mean (standard deviation) or n (%) depending on the type of variable.

### Model development and performance

The longitudinal and survival submodels are summarised in tables2  3 respectively. In the longitudinal submodel, the number of seizures in the 3 months before pregnancy, and younger age at first seizure were associated with lower serum lamotrigine concentrations. In the survival submodel, the number of non-tonic-clonic seizures in the 3 months before pregnancy (HR 1.022, 95% CI 1.007 to 1.038, p=0.004) and baseline lamotrigine dose (HR 1.004, 95% CI 1.002 to 1.006, p<0.001) were associated with increased risk of seizure occurrence in pregnancy or up to 6 weeks postpartum.

**Table 2:** Longitudinal submodel to predict serum lamotrigine levels during follow-up.

	Univariable models		Multivariable model	
**Predictors**	**Estimated coefficient (β) (95% CI**)	**p-value**	**Estimated coefficient (β) (95% CI**)	**p-value**
Time* (days)	0.009 (0.007 to 0.011)	<0.001	0.002 (0.002 to 0.003)	<0.001
Number of pre-pregnancy tonic-clonic seizures	0.364 (0.088 to 0.639)	0.010	**0.090 (0.005 to 0.175**)	**0.038**
Number of pre-pregnancy non-tonic-clonic seizures	0.017 (-0.048 to 0.014)	0.278	**−0.011 (-0.020 to −0.002**)	**0.018**
Age at first seizure	−0.050 (-0.085 to −0.014)	0.007	**−0.018 (-0.029 to 0.007**)	**0.001**
Maternal age	−0.009 (-0.068 to 0.049)	0.748	–	–
Seizure-related hospital admission in previous pregnancy	0.498 (-0.501 to 1.498)	0.326	–	–
Mental illness	0.169 (-0.649 to 0.986)	0.685	–	–
Learning difficulties	0.866 (-0.973 to 2.706)	0.354	–	–
Baseline seizure classification		0.056		
Non-tonic-clonic	*Reference*			
Tonic-clonic	−0.250 (-0.783 to 0.282)		–	–
Unspecified only	−1.645 (-3.083 to −0.208)		**–**	–
Baseline daily dose of lamotrigine	0.010 (0.008 to 0.011)	<0.001	**–**	–
Gestational age at baseline	−0.073 (-0.135 to −0.012)	0.019	−0.002 (-0.005 to<0.001)	0.060

Number of pre-pregnancy seizures as occurring in the three months prior. Model fitted with n = 183. CI: confidence interval.

Bold values indicate p<0.05.

* interval between date of study inclusion and serum sample measurement.

**Table 3:** Survival submodel to predict risk of seizure occurrence.

Univariable models		Multivariable model	
**Predictors**	HR (**95% CI**)	**p-value**	HR (**95% CI**)	**p-value**
Number of pre-pregnancy tonic-clonic seizures	1.211 (1.064 to 1.378)	0.004	1.064 (0.928 to 1.220)	0.376
Number of pre-pregnancy non-tonic-clonic seizures	1.026 (1.011 to 1.041)	<0.001	**1.022 (1.007 to 1.038**)	**0.004**
Baseline daily dose of lamotrigine	1.004 (1.003 to 1.006)	<0.001	**1.004 (1.002 to 1.006**)	**<0.001**
Age at first seizure	0.966 (0.930 to 1.004)	0.077	–	–
Maternal age	0.981 (0.919 to 1.004)	0.560	–	–
Gestational age at baseline	1.025 (0.957 to 1.098)	0.482	–	–
Seizure-related hospital admission in previous pregnancy	1.446 (0.571 to 3.661)	0.436	–	–
Seizure classification	0.271
Non-tonic-clonic	*Reference*		–	–
Tonic-clonic	0.611 (0.330 to 1.129)	–	–
Unspecified only	0.956 (0.228 to 4.015)	–	–
Mental illness (yes)	1.206 (0.511 to 2.846)	0.668	–	–
Learning difficulties (yes)	1.058 (0.146 to 7.680)	0.955	–	–

Number of pre-pregnancy seizures as occurring in the 3 months prior. Model fitted with n=183.

Bold values indicate p<0.05.

In the joint model, combining the longitudinal and survival submodels (table 4), pre-pregnancy tonic-clonic and non-tonic-clonic seizures, and age at first seizure remained associated with changes in serum lamotrigine concentrations during pregnancy. In the survival component, baseline lamotrigine dose (HR 1.005, 95% CI 1.003 to 1.007, p<0.001) remained significantly associated with seizure risk. Lamotrigine serum concentrations measured during pregnancy were not associated with seizure occurrence (HR 0.421, 95% CI 0.157 to 1.133, p=0.087). Examination of residual plots confirmed that model assumptions were met (data not shown).

**Table 4:** Results of the joint longitudinal and survival model to predict risk of seizure occurrence.

Longitudinal process (serum lamotrigine level)	Estimated coefficient (β)	95% CI	p-value
Time* (days)	0.002	0.002 to 0.003	<0.001
Gestational age at baseline	−0.002	−0.005 to 0.001	0.132
Number of pre-pregnancy tonic-clonic seizures	**0.097**	**0.012 to 0.183**	**0.026**
Number of pre-pregnancy non-tonic-clonic seizures	**−0.011**	**−0.020 to −0.002**	**0.014**
Age at first seizure	**−0.017**	**−0.028 to −0.006**	**0.003**
Event process (risk of seizure occurrence)	HR	**95%** CI	**p-value**
Number of pre-pregnancy tonic-clonic seizures	1.126	0.966 to 1.314	0.129
Number of pre-pregnancy non-tonic-clonic seizures	1.011	0.992 to 1.030	0.258
Baseline daily dose of lamotrigine (mg)	**1.005**	**1.003 to 1.007**	**<0.001**
Serum lamotrigine level (mg/l)	0.421	0.157 to 1.133	0.087

Number of pre-pregnancy seizures as occurring in the 3 months prior. Model fitted with n=183.

Bold values indicate p<0.05.

* interval between date of study inclusion and serum sample measurement.

### Internal validation and discrimination

**Figure 1:**
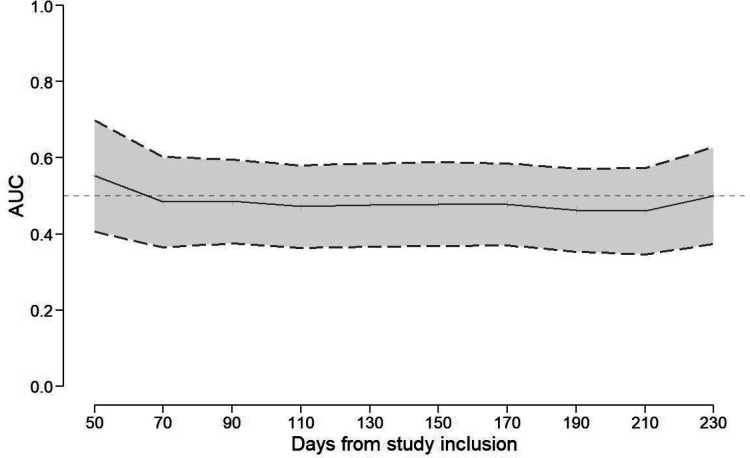
Time-dependent area under the receiver operator curve (AUC) with 95% confidence interval at multiple time points.

Internal validation using five-fold cross-validation showed the model had limited discrimination. The area under the curve remained close to 0.5 throughout pregnancy, indicating that longitudinal lamotrigine concentrations had no predictive ability for seizure occurrence (figure 1).

Analyses using relative lamotrigine concentrations, expressed as absolute changes from baseline and as ratios, did not improve model performance (data not shown).

## Discussion

In pregnant women with epilepsy taking lamotrigine at baseline, serial serum lamotrigine concentrations measured during pregnancy did not predict seizure occurrence, and baseline lamotrigine daily dose was the only independent predictor of seizure risk.

To our knowledge, this is the first study to apply joint longitudinal survival modelling to serial lamotrigine concentrations in pregnancy. We used prospectively-collected high-quality data from a large, multicentre cohort with serial serum measurements across pregnancy and the postnatal period. By building directly on the previously validated EMPiRE prediction model, we assessed whether drug levels add value beyond established clinical predictors.^2 21 29^ Our analyses primarily used absolute serum concentrations with monthly sampling, and we also tested relative changes from baseline and target concentration ratios, as some studies suggest these may better predict seizures.^15 30^ However, these measures did not improve model performance. Missing values of predictors were dealt with by multiple imputation thereby avoiding loss of useful information, and cross-validation with residual checks reduced the risk of overfitting.^31^ By applying joint modelling, we were able to account for the longitudinal trajectory of lamotrigine levels rather than assuming static values as in conventional time-to-event analyses.

Our study has limitations. This was a secondary analysis of EMPiRE trial participants with available lamotrigine measurements, and the findings should therefore be interpreted in this context. Our analysis was restricted to lamotrigine, so the findings may not generalise to other medications with different pharmacokinetic patterns. However, lamotrigine is one of the most prescribed antiseizure medications in pregnancy, so our findings remain relevant to clinical care. Our sample size limited subgroup or interaction testing, so we were unable to assess whether predictive value differed between women on monotherapy versus polytherapy, or between those in the therapeutic versus clinical features arm of the EMPiRE trial. Another limitation of this study is that we did not explicitly evaluate whether predefined threshold declines in lamotrigine concentrations relative to an individual reference level were associated with seizure risk. In addition**,** most seizures occurred early in pregnancy, leaving few women with repeat serum measurements before the first event. This limited the amount of longitudinal data available and reduced the model’s ability to assess how changes in serum drug levels related to seizure risk. Pre-pregnancy antiseizure medication serum levels were rarely available in the EMPiRE trial, so the first level obtained during pregnancy was used as baseline. This may have underestimated reductions in lamotrigine concentrations, but reflects routine clinical practice where early pregnancy levels are used as the reference. Lamotrigine dose adjustments during pregnancy were also not included as time-varying covariates in the joint model. The serum lamotrigine concentration, modelled as the longitudinal biomarker, reflects the combined effect of pharmacokinetic changes and any dose adjustments made during pregnancy, so it is not possible to disentangle these effects. Although missing baseline data were handled using multiple imputation, the presence of missing data remains a potential source of uncertainty, particularly for the variable capturing tonic-clonic seizures in the 3 months before pregnancy, where missingness exceeded 10%. Although internally validated, external validation is required before implementing these findings into routine practice.

Earlier studies report that pregnancy increases drug clearance and lowers serum concentrations, which might worsen seizure control.^1532^,^34^ Small, mainly single-centre studies reported that seizures were more likely when lamotrigine levels fell to about two-thirds of a woman’s pre-pregnancy level.^335^,^37^ The studies varied in how seizures were recorded and how concentrations were measured but provided the physiological rationale for therapeutic drug monitoring and informed guidance recommending regular measurement of lamotrigine concentrations in pregnancy with dose adjustment to maintain pre-pregnancy levels.^16 18^

More recent, larger studies have not confirmed an association between falling drug levels and seizure worsening.^20 38^ In the EMPiRE trial, routine therapeutic drug monitoring in pregnancy did not reduce seizure occurrence compared with clinical monitoring but increased fetal exposure to antiseizure medication.^20^ Large prospective cohorts, including the MONEAD study, have shown that serum concentrations of lamotrigine and levetiracetam fall during pregnancy, but seizure frequency does not increase in most women.^38^ Many women remain seizure-free despite large reductions in serum concentrations while some experience breakthrough seizures with only small changes in levels.^38^ This variability suggests that the link between serum concentration and seizure control may not be straightforward and that clinical context matters more than absolute drug levels. In the EMPiRE trial, higher baseline lamotrigine dose in women with seizures likely reflects greater underlying epilepsy severity and clinical indication for higher dosing.

Studies also show that pharmacokinetic changes during pregnancy differ between drugs.^13 39^ Lamotrigine clearance can more than double in some women by the third trimester while changes in levetiracetam and carbamazepine are smaller.^13 39^ This may also explain why guidance has focused mainly on lamotrigine and why studies of other drugs have not found consistent patterns between serum levels and seizure control.^13 18^

Larger studies with more frequent sampling may help clarify short-term changes in drug levels and how these relate to seizures. Research should also explore how clinical factors such as adherence, sleep and hormonal changes interact with pharmacokinetic changes to influence seizure risk. Future research is needed to determine whether therapeutic drug monitoring benefits certain subgroups, such as women taking more than one medication or with harder-to-control epilepsy. External validation of our model in other populations and with other antiseizure medications is also needed to confirm its generalisability and refine risk thresholds. Trials comparing standard care with approaches guided by validated, individualised risk models would show whether these strategies improve clinical outcomes. Research should also consider possible secondary benefits of therapeutic drug monitoring, such as reassurance, adherence or engagement with care and assess their value alongside clinical outcomes.

Our study shows that regular monitoring of lamotrigine levels in pregnancy does not improve seizure control. Clinical management should instead be guided primarily by seizure history, adherence and overall clinical assessment. Drug level monitoring may still be appropriate in some situations, such as when adherence is uncertain, when vomiting limits absorption, when another medicine is added that could alter lamotrigine levels, or if toxicity is suspected.^40^

Effective care depends on early identification of women at increased risk of seizures, coordination between obstetric and neurology services, and regular clinical review throughout pregnancy. Individualised counselling about seizure risks and medication adjustment is central to safe care. Dose adjustments should be guided by clinical judgement rather than absolute serum concentrations and therapeutic drug monitoring used only when the results are likely to change management. Current guidance recommending routine monitoring, particularly for lamotrigine, should be reconsidered given its limited clinical value and potential for increased fetal exposure. Routine monitoring requires resources and may detract from clinical assessment that better informs seizure management. Policy should promote a selective, needs-based approach that prioritises clinical evaluation and shared decision-making between clinicians and women.

In pregnant women with epilepsy taking lamotrigine, changes in serum concentrations during pregnancy were not associated with seizure occurrence. Baseline lamotrigine dose was the only independent predictor of seizure risk. We found limited evidence to support routine monitoring of lamotrigine levels for predicting seizures.

## Supplementary material

10.1136/bmjno-2026-001547 online supplemental file 1

## Data Availability

Data are available upon reasonable request.
