# Supplementary material for: Lamotrigine serum concentrations as longitudinal biomarkers for seizure risk prediction in pregnant women with epilepsy: a secondary analysis of the EMPiRE study
Source: BMJ Neurol Open. 2026 Jun 22;8(1):e001547. doi: 10.1136/bmjno-2026-001547 (PMC13289018; doi:10.1136/bmjno-2026-001547)
Supplement: online supplemental file 1 [file bmjno-8-1-s001.docx]

**Appendix A**

The first step is to develop the longitudinal model by fitting a linear mixed effect model. In our study, this implies describing the evolution of the lamotrigine values adjusted for baseline covariates. This approach assumes a normal distribution for the marker. Several transformations of the model have also been considered to check how potential assumption violations could affect the model's performance.

In the second step, a Cox regression model is developed to analyse time to first seizure. The set of independent variables to be included in the multivariable submodels was selected first adjusting univariate regression models and retaining those with a statistically significant association (p<0.15).

Finally, the information generated by both models is combined. The joint model (JM) is obtained using the JM package in R (R Core Team, 2024; Rizopoulos, 2022), assuming spline approximated baseline risk function. A diagnostic of the model was performed for both the longitudinal and the time-to-event models, based on the appropriate residuals. Multicollinearity between independent variables was also checked.

Details of the submodels are below:

The longitudinal submodel is:

where $\beta_{0}+b_{0i}$ represents the intercept, consisting of a fixed effect ($\beta_{0}$) and a random effect ($b_{0i}$) which accounts for inter-individual variability; $\left( \beta_{1}+b_{1i} \right)$ is the slope associated with time $t$, where $\beta_{1}$is the fixed component and $b_{1i}$ is the random component, allowing the effect of time to vary across individuals;​ $\varepsilon_{i}(t)$ represents the residual error for the $i$-th individual at time $t$ and $\sigma^{2}$ is the common error variance.

And the survival submodel:

where the baseline hazard function, $h_{o},$ is assumed a spline function; the coefficients $\gamma_{1},\gamma_{2},\gamma_{3}$, represent the effect of the baseline risk factors, while the term $\alpha$ represents the influence of the temporal evolution of the *LTG*.

**Appendix B**

Flow diagram of EMPiRE participants that contributed to model development.

Pregnant women with epilepsy eligible for EMPiRE trial **(n=560)**

Not using lamotrigine **(n=254)**

First lamotrigine measurement after first seizure **(n=99)**

Have at least one lamotrigine serum measurement before first seizure or both six weeks postpartum **(n=183)**

No baseline or follow-up serum lamotrigine data **(n=24)**

On lamotrigine monotherapy or polytherapy **(n=306)**

Have baseline and follow-up serum lamotrigine data **(n=282)**
